# Project CHARIOT: study protocol for a hybrid type 1 effectiveness-implementation study of comprehensive tele-harm reduction for engagement of people who inject drugs in HIV prevention services

**DOI:** 10.1186/s13722-024-00447-9

**Published:** 2024-03-25

**Authors:** Tyler S. Bartholomew, Marina Plesons, David P. Serota, Elizabeth Alonso, Lisa R. Metsch, Daniel J. Feaster, Jessica Ucha, Edward Suarez, David W. Forrest, Teresa A. Chueng, Katrina Ciraldo, Jimmie Brooks, Justin D. Smith, Joshua A. Barocas, Hansel E. Tookes

**Affiliations:** 1 Division of Health Services Research and Policy, Department of Public Health Sciences, University of Miami Miller School of Medicine, 1120 NW 14th St, Miami, FL 33136 USA; 2 Division of Infectious Diseases, Department of Medicine, University of Miami Miller School of Medicine, Miami, FL USA; 3 Department of Sociomedical Sciences, Mailman School of Public Health, Columbia University, New York, NY USA; 4 Biostatistics Division, Department of Public Health Sciences, University of Miami Miller School of Medicine, Miami, FL USA; 5 Department of Psychiatry, University of Miami Miller School of Medicine, Miami, FL USA; 6 Department of Anthropology, University of Miami, Miami, FL USA; 7 Department of Population Health Sciences, Spencer Fox Eccles School of Medicine at the University of Utah, Salt Lake City, Utah USA; 8 Divisions of General Internal Medicine and Infectious Diseases, Department of Internal Medicine, University of Colorado School of Medicine, Aurora, CO USA

## Abstract

**Background:**

People who inject drugs (PWID) remain a high priority population under the federal Ending the HIV Epidemic initiative with 11% of new HIV infections attributable to injection drug use. There is a critical need for innovative, efficacious, scalable, and community-driven models of healthcare in non-stigmatizing settings for PWID. We seek to test a *Comprehensive-TeleHarm Reduction (C-THR)* intervention for HIV prevention services delivered via a syringe services program (SSP).

**Methods:**

The CHARIOT trial is a hybrid type I effectiveness-implementation study using a parallel two-arm randomized controlled trial design. Participants (i.e., PWID; n = 350) will be recruited from a syringe services program (SSP) in Miami, Florida. Participants will be randomized to receive either *C-THR* or non-SSP clinic referral and patient navigation. The objectives are: (1) to determine if the *C-THR* intervention increases engagement in HIV prevention (i.e., HIV pre-exposure prophylaxis; PrEP or medications for opioid use disorder; MOUD) compared to non-SSP clinic referral and patient navigation, (2) to examine the long-term effectiveness and cost-effectiveness of the *C-THR* intervention, and (3) to assess the barriers and facilitators to implementation and sustainment of the *C-THR* intervention. The co-primary outcomes are PrEP or MOUD engagement across follow-up at 3, 6, 9 and 12 months. For PrEP, engagement is confirmed by tenofovir on dried blood spot or cabotegravir injection within the previous 8 weeks. For MOUD, engagement is defined as screening positive for norbuprenorphine or methadone on urine drug screen; or naltrexone or buprenorphine injection within the previous 4 weeks. Secondary outcomes include PrEP adherence, engagement in HCV treatment and sustained virologic response, and treatment of sexually transmitted infections. The short and long term cost-effectiveness analyses and mixed-methods implementation evaluation will provide compelling data on the sustainability and possible impact of *C-THR* on comprehensive HIV prevention delivered via SSPs.

**Discussion:**

The CHARIOT trial will be the first to our knowledge to test the efficacy of an innovative, peer-led telehealth intervention with PWID at risk for HIV delivered via an SSP. This innovative healthcare model seeks to transform the way PWID access care by bypassing the traditional healthcare system, reducing multi-level barriers to care, and meeting PWID where they are.

*Trial Registration*: ClinicalTrials.gov NCT05897099. Trial registry name: Comprehensive HIV and Harm Prevention Via Telehealth (CHARIOT). Registration date: 06/12/2023.

## Introduction

### Background and rationale

Injection drug use (IDU) continues to be an important mode of transmission for HIV infection, and people who inject drugs (PWID) are a high priority population under the United States (US) Ending the HIV Epidemic initiative [1]. In 2020, 11% of new HIV infections were attributable to IDU [2]. IDU has led to multiple recent outbreaks of HIV in the US, driven primarily by the ongoing stimulant and opioid crises and exacerbated by the COVID-19 pandemic, creating an obstacle to meeting Ending the HIV Epidemic goals [3–8]. The Ending the HIV Epidemic initiative has identified evidence-based interventions including rapid HIV testing, antiretroviral therapy (ART), comprehensive syringe services programs (SSPs), and pre-exposure prophylaxis (PrEP) that need to be implemented, scaled, and sustained within communities most affected by HIV. To maximize the effectiveness of these interventions among PWID, comprehensive healthcare models must be developed, tested, and deployed where PWID are, in comfortable, non-stigmatized environments that simultaneously address a key driver of HIV, namely untreated substance use disorder (SUD).

PWID often experience pervasive discrimination, stigma, degradation and social disadvantage when accessing the traditional healthcare system, leading to almost universal poorer health outcomes compared to non-PWID [9–17]. However, one long-standing, trusted, and frequented institution for PWID are SSPs. Decades of research have highlighted their effectiveness in HIV prevention [18–23] and, more recently, their ability to implement HIV testing [24], PrEP [25], medications for opioid use disorder (MOUD) [26], and overdose education and naloxone distribution [27]. Increased access to MOUD has been shown to facilitate positive care outcomes among people with opioid use disorder (OUD) [28, 29], and SSPs have been shown to be ideal venues to offer PrEP [30] and buprenorphine [31–33] to further mitigate HIV risk and acquistion. However, initiating and retaining PWID on PrEP and MOUD remains a major implementation challenge, limiting our ability to meet Ending the HIV Epidemic targets. Multiple studies have demonstrated significant gaps in the PrEP cascade for PWID, leading to extremely low uptake, initiation, and persistence on PrEP (0–3%) [34–36]. Likewise, the OUD care cascade has pervasive gaps, with low rates of OUD screening, MOUD initiation, and MOUD retention among PWID [37]. Questions remain on how to most effectively implement sustainable and scalable integrated HIV prevention and OUD care models in SSPs for PWID.

We developed, refined, and pilot tested *tele-harm reduction (THR)*: a telehealth-based, multicomponent care model for PWID with HIV implemented at the IDEA Miami SSP. *THR* integrates multilevel stigma reduction [38, 39] and harm reduction frameworks [40] to provide access to HIV care through telehealth in an SSP setting that is supported by on-site peer-driven systems of care to enhance treatment initiation and adherence. *THR* has preliminarily demonstrated high acceptability and promising outcomes with 78% of PWID with HIV achieving viral suppression at 6 months post-enrollment, significantly higher than current national estimates which show 56.9% of PWID are virally suppressed [2, 40–42]. Our interdisciplinary research team is currently testing the efficacy of the *THR* model for HIV viral suppression in a randomized controlled trial (RCT) under a National Institute on Drug Abuse Avenir Award (DP2DA053720, PI: Tookes).

Building upon this work of *THR* for HIV treatment in PWID and in pursuit of a status neutral [43] intervention for comprehensive HIV care, we developed *comprehensive tele-harm reduction (C-THR)* to address HIV prevention in this high priority community. The overall goal of the CHARIOT trial is to compare the efficacy of the *C-THR* intervention in engaging PWID in HIV prevention services (i.e., PrEP and/or MOUD) to current standard of care (i.e., non-SSP clinic referral and patient navigation) using a parallel two-arm RCT, and evaluate the process of implementation consistent with the aims of a hybrid type I effectiveness-implementation study [44].

### Objectives

The first objective of the study is to compare the efficacy of the *C-THR* intervention for engagement in HIV prevention services (i.e., PrEP and MOUD) versus non-SSP clinic referral and patient navigation, with the co-primary outcome the time averaged proportion of participants engaged with PrEP or MOUD across follow-up at 3, 6, 9 and 12 months. For PrEP, engagement is confirmed by tenofovir on dried blood spot or cabotegravir injection within the previous 8 weeks. For MOUD, engagement is defined as screening positive for norbuprenorphine or methadone on urine drug screen (UDS); or extended release buprenorphine or naltrexone injection within the previous 4 weeks. The primary hypothesis is that C-THR will be superior to non-SSP clinic referral and patient navigation in promoting engagement of PWID in HIV prevention.

In relation to the primary objective, we will also measure differences between study arms in several secondary outcomes including syringe coverage, PrEP adherence, engagement in hepatitis C virus (HCV) treatment, rates of HCV cure, treatment of sexually transmitted infections (STIs), time to injection-related harm and number of injection-related harms. Participants in the C-THR arm will have peer-facilitated enhanced access to a physician via a telehealth platform, phlebotomy at both fixed and mobile sites, peer-facilitated medication management (e.g., storage in pill lockers at SSP, weekly delivery), appointment reminders, and mental health counseling. Participants assigned to the non-SSP clinic referral and patient navigation arm will be linked to a community health center or primary care clinic (e.g., Federally Qualified Health Center) by SSP staff as is the current standard of care with patient navigation for the first clinic visit.

The second objective of the study is to estimate the short- and long-term clinical- and cost-effectiveness of *C-THR* versus non-SSP clinic referral and patient navigation. The economic analysis will follow best practices for conducting economic analyses alongside clinical trials [45–47]. The long-term clinical and cost-effectiveness will utilze a validated simulation model [48, 49]. The REDUCE model was designed to simulate the natural history of IDU and understand how both hospital- and community-based interventions designed to *reduce* injection frequency and *reduce* risky injection behaviors (e.g., decrease needle reuse) may improve long-term outcomes.

The third objective of the study is to assess the implementation context (ie. barriers and facilitators) to successful *C-THR* implementation in an SSP setting and evaluate the process of implementation over the course of the study. Our mixed-methods implementation evaluation will leverage the Practical, Robust Implementation and Sustainability Model (PRISM) to understand how external and internal contextual factors influence reach, implementation, and maintainence of *C-THR.*

### Trial design

The CHARIOT trial is a hybrid type 1 effectiveness-implementation study using a non-blinded RCT design with two parallel treatment arms. Participants who are PWID and who have a non-reactive result on rapid HIV test will be randomly assigned to either *C-THR* with telehealth services for HIV prevention (syringes, PrEP, MOUD) and treatment of HCV, STIs, and skin and soft tissue infection (SSTIs) that can be accessed wherever the patients are (e.g., SSP, mobile unit, outreach at homeless encampment, shelter, or patient home); or, to non-SSP clinic referral with support from a patient navigator for first clinic visit for PrEP, MOUD, treatment of HCV, STIs, and SSTIs. Randomization will occur by permuted block with stratification for site (fixed vs. mobile) and OUD (yes vs. no). An implementation process evaluation and cost-effectiveness analysis will also be performed. The study protocol (version 1.0) follows the Standard Protocol Items: Recommendations for Interventional Trials (SPIRIT) Statement.

## Methods: participants, interventions and outcomes

### Study setting

Miami is an ideal city for studying *C-THR* in the context of an SSP. The IDEA Miami SSP was authorized in 2016 as the first legal SSP in Florida. There are currently over 2200 PWID enrolled, with 9% testing reactive for HIV (10.4% at the mobile site, 8.2% at the fixed site) and 41% for HCV (51.1% at the mobile site, 39.0% at the fixed site) through our routine every 3 months opt-out HIV/HCV screening algorithm [24]. Miami-Dade County is a high priority county under the Ending the HIV Epidemic initiative [1] and has a high prevalence of PWID with estimates ranging from 7607 to 10,529 [50, 51]. IDEA Miami is housed within an academic medical center (the University of Miami) in partnership with one of the largest safety-net hospitals in the country (Jackson Health System), and is supported by major research centers including the NIH-funded Miami Center for AIDS Research and NIH-funded Center for HIV and Research in Mental Health. IDEA Miami exchanges over 30,000 syringes per month and operates one fixed site and five mobile sites in both the downtown area as well as rural agricultural areas in Miami-Dade County.

### Eligibility criteria

Eligibility criteria include the following: (1) age 18 or older; (2) able to speak English or Spanish; (3) willing and able to sign informed consent, provide locator information and medical records release; (4) non-reactive result on rapid HIV test; (5) use of SSP to exchange syringes two times in the past 3 months; and (6) planning to stay in the area for 12 months. Of note, pregnant people are eligible for enrollment in this trial but will not be purposely recruited.

Exclusion criteria include the following: (1) PWID with HIV; (2) currently on MOUD; (3) currently on PrEP; (4) principal or site investigator discretion; (5) currently in prison or jail; and (6) current enrollment in NIDA Clinical Trials Network (CTN) 121 which is a study that our team is leading in Miami to test a comprehensive, integrated hospital-based intervention to address concurrent treatment and follow-up support for PWID with severe infections; (7) current enrollment in M2HepPrEP (R01 DA045713) [52] which is an integrated Hepatitis C and HIV prevention study that our team is completing this year; (8) hepatitis B surface antigen positive; (9) receipt of *THR* in the previous 3 months; and (10) signs or symptoms of acute HIV infection. Investigator discretion could include serious medical, psychiatric, or co-occurring SUD that acutely requires a higher or different level of care including the following: (1) disabling or terminal medical illness (e.g., decompensated heart failure); (2) severe, untreated or inadequately treated psychiatric condition (e.g., active psychosis); (3) in need of medical detoxification for severe alcohol, benzodiazepine, or other depressant or sedative hypnotic use; (4) suicidal intent or plan; and (5) homicidal ideation.

### Who will obtain informed consent?

Study staff including the study coordinator, research assistant, and peer counselor will recruit participants and obtain informed consent. Screening informed consent will be obtained at the SSP fixed site or mobile sites after a participant has screened non-reactive for HIV. The main informed consent will be obtained after a participant is determined to be eligible for the study.

### Additional consent provisions for collection and use of participant data and biological specimens

Individuals who meet preliminary screening criteria (i.e., that the participant is a current SSP client with a non-reactive HIV rapid test) will be requested to complete the screening informed consent process including medical record release, a fourth generation HIV test, hepatitis serologies and HCV RNA, 3-site gonorrhea and chlamydia testing, syphilis screening, comprehensive metabolic panel, complete blood count, urine drug screen, urine pregnancy test (if applicable), and a urine tenofovir test. Consent for screening will be obtained by study staff using an institutional review board (IRB)-approved written consent form.

For individuals who meet the full eligibility criteria and would like to enter the study, informed consent will be obtained by study staff using an IRB-approved written consent form in English or Spanish agreeing to the possibility of telehealth with wraparound services for possible PrEP, MOUD, and treatment of HCV, STIs or SSTIs. They will also be required to provide locator information including phone number, email, social media accounts (e.g., Facebook, Instagram, Linkedin) and friend/family contact information and medical records release. Participants will receive a copy of all consent documents.

No additional studies are planned at this time.

## Interventions

### Explanation for the choice of comparators

The CHARIOT study is proposed as an efficacy trial because the study team has already demonstrated the feasibility and acceptability of telehealth for rapid antiretroviral (ART) initiation among PWID and is currently conducting the T-SHARP trial to determine the efficacy of telehealth for HIV care among PWID [40]. *C-THR* will be based out of the IDEA Miami SSP due to its established relationship of trust within the PWID community, and all study participants will be registered clients of the SSP. The study will be a randomized 2-arm trial with participants randomized to either C-*THR* with enhanced telehealth services for PrEP, MOUD, and treatment of HCV, STI or SSTI that can be accessed where the patients are (e.g., SSP, mobile unit, outreach at homeless encampment, or patient home) or to non-SSP clinic referral with a patient navigator. Recruitment will take place at the IDEA Miami SSP and participants will be assessed at 3, 6, 9, and 12 months following randomization to measure PrEP engagement (tenofovir on DBS or abstraction of cabotegravir injection records) or MOUD engagement (norbuprenorphine or methadone on UDS; or abstraction of naltrexone or buprenorphine injection records), as well as syringe coverage, PrEP adherence, HCV treatment engagement, HCV cure, time to injection-related harm and number of injection-related harms. Quantitative assessments will help estimate predictors of engagement in HIV prevention services via the C-*THR* intervention package. Just as with our T-SHARP trial, it is expected that syndemic factors [53, 54] such as unstable housing and/or food insecurity will impact engagement in HIV prevention services across time points and that the C-*THR* intervention will perform better at mitigating the effects of these potential confounders.

### Intervention description

#### C-THR intervention arm

Component 1 of the *C-THR* intervention utilizes telehealth technology facilitated by a peer counselor to connect participants to on-demand visits with a study physician and clinical psychologist. Technology is available wherever the participant is located and prefers engagement (e.g., SSP, home, shelter, encampment) via a HIPAA-compliant videoconferencing application, iPads, and headphones for privacy. All study physicians practice medicine through a harm reduction lens and are experienced in HIV prevention and the treatment of SUD. A call schedule across the study physicians will support on-demand access for participants. By incorporating enhanced access to a clinical psychologist, the *C-THR* intervention will also be capable of addressing the estimated 40% of people with SUD who have co-occurring mental health disorders [55] (e.g., post-traumatic stress disorder, depression and anxiety) as well as the participants who have stimulant use disorders. The psychologist will formulate a treatment plan and visit frequency with study physicians as determined by clinical indication(s), and will provide ongoing services including talk therapy and psychotropic medications to address concurrent mental health disorders. This essential component will allow for an evidence-based approach to co-occurring stimulant use disorders which are prevalent in the Miami PWID community [56] to augment emerging pharmacotherapies [57–60].

On-site, culturally appropriate blood draws will be performed at the SSP fixed or mobile sites by a medical assistant/phlebotomist and sent to the lab for testing. Blood will be sent to LabCorp to measure creatinine, and to the Department of Health to be tested for HIV, viral hepatitis, and STIs. The medical assistant/phlebotomist will swab the participants’ throats to test for gonorrhea and chlamydia and will instruct the participants on how to collect a self-swab for rectal gonorrhea and chlamydia in a private bathroom. Urine will be collected for gonorrhea and chlamydia screening, as well. Finally, point-of-care urine tests will assess the presence of buprenorphine or tenofovir.

Component 2 of the *C-THR* intervention utilizes SSP-based peer counselors who will work with the physicians and clinical psychologist to evaluate the participants and encourage them to initiate and remain in care using motivational interviewing based techniques. If indicated and desired, PrEP, MOUD, direct-acting antivirals (DAA) for HCV, and/or antibiotics for STI/SSTI will be prescribed by the physician. The study will not directly fund medications; rather, peer navigators and physicians will assist in securing access to therapies using the participant’s health insurance, county health system indigent care funds, or other available grants and sources. Prescribed medications will be picked up from the pharmacy by the peer counselor, or, in the case of antibiotics, immediately dispensed from the SSP. Participants will enroll in a weekly medication management schedule with medication storage via pill lockers available at the SSP. Participants will be able to choose between accessing their medications at the SSP or having them delivered by the peer counselor during routine engagement visits. Peer counselors will explore barriers to medication adherence with a focus on the importance of adherence and safe injection practices for positive health outcomes and will work to enhance participants’ self-efficacy and support overall wellbeing. Intensive wrap-around support from the peer counselors and digital technology will help us assist the participants in other domains such as housing, food insecurity, and insurance (essential to cover the cost of PrEP/DAA) and provide an opportunity for routine motivational interviewing from the integrated care team. Peer counselors will ideally be persons with the lived experience of SUD, mental health disorders, and/or trauma. All *C-THR* team interactions with participants will be culturally sensitive, non-stigmatizing and non-judgmental, grounded in respect and mutual aid. *C-THR* also provides needs-based access to syringes and naloxone alongside the delivery of PrEP and MOUD services.

Bringing the mobile telehealth technology along with the exchange of syringes to participants will allow us to overcome previously reported barriers to the use of telehealth among PWID, including lack of access to mobile phones [61–63]. A limitation to the intervention is that not all medical care can be delivered remotely via telehealth. For medical care that cannot be offered at the IDEA Miami SSP or remotely in the *C-THR* arm (e.g., x-ray, dental extraction), peer counselors will navigate participants to appropriate free care within the academic safety-net health systems. Not every service component of the intervention will be delivered via telehealth (e.g., provision of syringes), and in-person engagement with the peer counselor is an essential component of the *C-THR* intervention.

The *C-THR* intervention manual will be adapted from the *THR* intervention manual currently being used in the T-SHARP trial via consultation with the IDEA Miami SSP staff, program participants and the Florida Harm Reduction Collective Community Advisory Board. Participants in the *C-THR* arm will have all intervention sessions (i.e., physician, psychologist and peer counselor encounters) audio recorded. Participants will be given the option to opt out of audio-recording.

At the end of the 12-month study period, participants randomized to the *C-THR* arm will be offered continuation in the *C-THR* intervention.

### Non-SSP clinic referral and patient navigation arm

Participants who are randomized to non-SSP clinic referral and patient navigation (i.e., the control condition) will receive the current linkage to care protocols at the IDEA Miami SSP including case management/social work services through our community engagement team. This team is comprised of case managers and social workers and provides the wraparound support needed to navigate the fragmented healthcare system in Miami. The team will assist participants in scheduling appointments at community health clinics including FQHCs or the safety-net Jackson Health System. The community engagement team provides active clinic referral—that is, a member of the team accompanies participants to their first clinic visit. The staff can provide transport in the mobile van or use ride-share (e.g., Lyft Healthcare) to actively link participants to registration, phlebotomy, and pharmacy services. This model of patient navigation has helped PWID achieve rapid HIV viral suppression at our SSP [7].

The community engagement team will work to link to non-SSP clinics that offer PrEP, MOUD, treatment for HCV/STI/SSTI, and/or mental health services based on participant needs to promote parallel access to care between the two arms. Crossover could occur if participants in the *C-THR* group receive services at a community health center; however, in our experience, PWID receiving services at the IDEA Miami SSP rarely independently seek out services at community health centers. Conversely, telehealth could occur in the control arm, but this would not be facilitated in the specific non-stigmatizing environment of an SSP and peer counselors would not bridge the digital divide, facilitating and motivating care seeking behaviors in participants. We will include questions on follow-up assessments that will be able to ascertain crossover in use of services.

At the end of the 12-month study period, participants randomized to the non-SSP clinic referral and patient navigation arm will be offered entry into the *C-THR* intervention.

### Criteria for discontinuing or modifying allocated interventions

There will be no special criteria for discontinuing or modifying allocated interventions.

### Strategies to improve adherence to interventions

In order to assure fidelity to the C-*THR* intervention, all intervention sessions (i.e., physician, psychologist, and peer counselor encounters) will be audio recorded. Participants will be given the option to opt out of audio-recording. Ten percent of the audio-recorded intervention sessions will be reviewed by the study clinical supervisor(s) and intervention developers for implementation fidelity monitoring purposes and also to provide ongoing clinical feedback to the study staff.

### Relevant concomitant care permitted or prohibited during the trial

Participants must agree to the possibility of receiving telehealth-enhanced access to HIV prevention services via the IDEA Miami SSP. However, that does not preclude them from engaging with other HIV/AIDS service organizations, primary care providers, or substance use treatment programs for further support.

### Provisions for post-trial care

After study completion, participants in both arms will be offered the *C-THR* intervention.

### Outcomes

Table 1 shows the primary and secondary outcomes of objective 1. The co-primary outcomes will be engagement in HIV prevention via PrEP and/or via MOUD. PrEP engagement will be measured by intracellular levels of TFV-DP on DBS or abstraction of a cabotegravir injection in the previous 8 weeks. MOUD engagement will be measured by norbuprenorphine or methadone on UDS or abstraction of a naltrexone or buprenorphine injection in the previous 4 weeks.

**Table 1:** Primary and secondary outcomes for objective 1

	Definition	Measure
Primary outcome		
HIV Prevention via PrEP	Taking PrEP at 3-, 6-, 9-, and 12-month follow-up	Intracellular levels of tenofovir diphosphate (TFV-DP) by DBS or cabotegravir injection in previous 8 weeks by electronic health record abstraction
HIV Prevention via MOUD	Taking MOUD at 3-, 6-, 9-, and 12-month follow-up	Norbuprenorphine or methadone on UDS or naltrexone or buprenorphine extended-release injection in previous 4 weeks by electronic health record abstraction
Secondary outcomes		
Syringe coverage	Dispensing syringes to cover each injection event	Number of syringes distributed/(number of injections per day × days between exchanges) from SSP administrative data
PrEP Adherence	Taking PrEP at least 4 times per week	TFV-DP level of 700 fmol/punch on DBS for TDF [64]; TFV-DP level of 950 fmol/punch on DBS for TAF[65]
Engagement in HCV treatment	Receiving a prescription for DAA	DAA prescsiption confirmed on electronic health record abstraction
HCV cure (SVR 12)	Initiating HCV treatment and achieving undetectable HCV RNA PCR at least 12 weeks after completion of therapy	Negative HCV RNA PCR (viral load) at least 12 weeks post treatment completion
Treatment of STIs	Receiving treatment for STI after a positive screen	Prescription of appropriate antibiotics (may differ based on allergies) from electronic health record abstraction
Time to harm	Time to: (1) emergency department visit or hospitalization for injection-related infection or overdose; (2) incident HIV infection; (3) incident HCV infection; or (4) death from overdose	(1) Electronic health record abstraction; (2, 3) IDEA test results; (4) electronic health record abstraction or medical examiner database
Number of harms	Count of (1) emergency department visit or hospitalization for injection-related infection or overdose; (2) incident HIV infection; (3) incident HCV infection; or (4) death from overdose	(1) Electronic health record abstraction; (2, 3) IDEA test results (4) electronic health record abstraction or medical examiner database

Secondary analyses will evaluate positive health behaviors by counts per person per study arm. PrEP adherence will be defined as taking PrEP at least four times per week for men and at least 6 times per week for women as confirmed by TFV-DP levels on DBS. HCV treatment engagement will be defined as receipt of a prescription for DAA. HCV cure will be defined as sustained virologic response at least 12 weeks after completing DAA (SVR 12) and treatment of STIs is defined as receipt of the appropriate antibiotic treatment after diagnosis. Secondary outcomes will also evaluate negative health outcomes or harms by counts per person per study group. Emergency department visits or hospitalizations will be confirmed by participants’ consent for records release and abstraction from Miami area hospitals which have integrated electronic health records. Incident HIV and HCV infections (or reinfections) will be confirmed by positive 4th generation HIV antigen/antibody test and detected HCV RNA, respectively. Death from overdose will be confirmed by hospital record, Miami-Dade County Medical Examiner database search or direct inquiry with the medical examiner. In addition to counts, time to secondary outcomes, both positive and negative, will be compared by study arm.

For objective 2, the primary health economic outcomes will be: (1) implementation and ongoing management costs of the two healthcare models, and (2) short-term cost-effectiveness from the perspectives of the healthcare sector and society. The primary economic outcome will be the incremental cost-effectiveness ratio (ICER) from the healthcare sector and modified societal perspectives. The measure of effectiveness will be quality-adjusted life years (QALYs) and incident HIV infections averted.

For objective 3, the process evaluation will focus on contextual factors and strategies related to achieving the following implementation outcomes: (1) reach, (2) efficacy, (3) implementation (cost, fidelity, and adaptation), and 4) maintenance (Table 2). The “Reach” metric will be operationalized according to the RE-AIM guidelines [66] to understand the representativeness of individuals being recruited and enrolled into the trial compared to the general SSP population. “Implementation” outcomes that will be assessed include: acceptability (provider and staff level) according to the Theoretical Framework of Acceptability (TFA) [67] intervention appropriateness (provider and staff level), measured by the Intervention Appropriateness Measure (IAM) [68], implementation fidelity, and feasibility (participant level). The conceptual distinction of these outcomes are specified by Proctor’s taxonomy of implementation outcomes [69] (Table 2).

**Table 2:** Implementation outcomes for objective 3

Construct	Measure	Data source
RE-AIM implementation evaluation framework		
Reach	Proportion of eligible participants in SSP engaged in the intervention	SSP administrative records
Efficacy	Efficacy of the *C-THR* model on engagement in HIV prevention	Study Aim 1
Adoption	N/A	N/A
Implementation	PRISM Constructs (i.e. external environment, patient perspective)	Interviews with providers, SSP staff, and participants
Maintenance	Cost analysis, cost-effectiveness, long-term simulation modeling	Study Aim 2

### Participant timeline

**Fig. 1:**
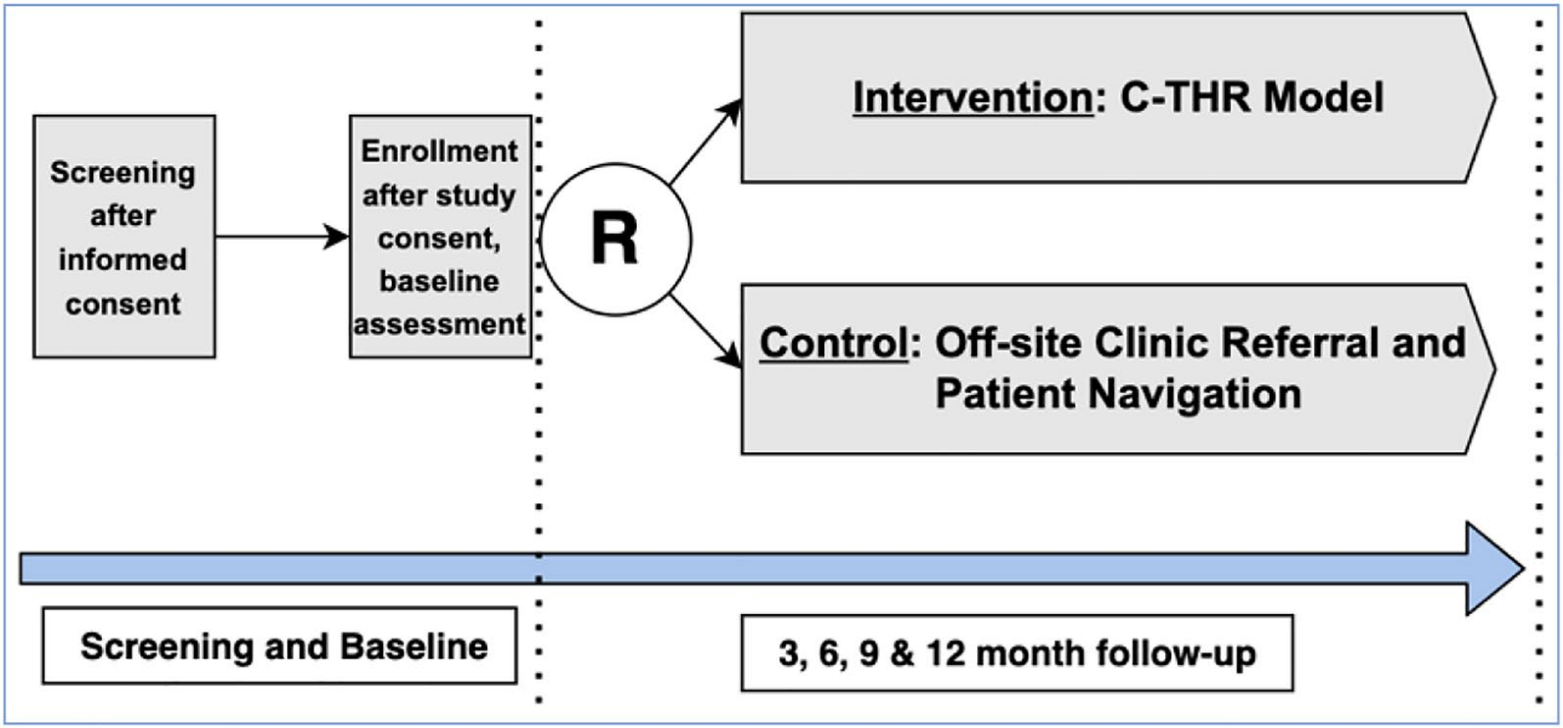
RCT study flow

Participant timeline is presented in Fig. 1.

### Sample size

Power analyses were generated using the two-group repeated proportions module in PASS 2020 [68] to compute minimum detectable effect sizes for the proposed primary analysis. The study will include 350 participants equally assigned to the intervention and control groups. We assume that as much as 10% of the sample may drop-out at each follow-up; however, we anticipate better retention due to SSP staff engagement in the study [69]. The expected sample sizes at the four follow-ups are thus 315, 284, 255, and 230. Using these sample sizes and assuming α = 0.025, power = 0.80, and that the proportion with PrEP or MOUD engagement will be 30% of the control arm, we computed the minimum detectable odds ratio (OR) 1.56 and absolute proportion difference (pdiff) of 0.101, assuming that all post-intervention time points are part of the contrast. We varied the within-subject correlation ρ from 0.10 to 0.80. Power for a binomial is lowest when the base-rate (control group) probability (P_0_) is near 50%.

### Recruitment

Participants will be recruited from the IDEA Miami SSP by our racially and ethnically diverse staff that includes peer counselors. For *C-THR*, individuals presenting to the IDEA Miami SSP who are interested in: (1) HIV/HCV testing; (2) PrEP; (3) MOUD; (4) STI screening; (5) treatment of HCV; and/or (6) assessment/treatment for SSTI will be asked if they would like to participate in a research study using technology for PWID-specific preventive healthcare.

1. Via Rapid Testing: The IDEA Miami SSP conducts routine HIV/HCV screening, with opt-out screening offered every 3 months. Individuals who test non-reactive for HIV will be asked if they would like to participate in a research study on using technology for HIV prevention.
2. Community Engagement Team Referral: The IDEA Miami SSP has a community engagement team to provide wrap-around support for PWID using the SSP. Any potential participant seeking the aforementioned medical services may be referred to the study coordinator.
3. Peer Referral: Randomized study participants will be provided an additional $50 incentive for each eligible participant referred to the study who is randomized up to four. Participants who screen fail will be provided an additional $50 incentive for each eligible participant referred to the study who is randomized up to eight.
4. Study Flyer: A flyer and palm card with contact information for the study coordinator will be posted at the SSP, on mobile units, and distributed through street outreach. The flyer may be posted on social media, as well as Reddit forums.
5. Hospitalized PWID: The Severe Injection Related Injury Team at Jackson Health System will refer potentially eligible participants. Participants will be provided a study flyer and referred to the IDEA Miami SSP to sign screening consent. Study procedures will be deferred until the patient is ambulatory at the SSP.

It is anticipated that throughout the recruitment period, encounters for routine HIV/HCV screening, PrEP, MOUD, STI screening/treatment, and/or assessment for SSTI will facilitate identification of 440 participants who could be eligible for the study. Of these, we expect 80% (n = 352) to be eligible for randomization.

For completing the screening process, including quantitative assessment and laboratory analysis, participants will receive $50 total. Participants in both arms will receive $50 per laboratory assessment (at 3, 6, 9, and 12 months) and $50 per quantitative assessment (at 0, 6, and 12 months). Fifteen participants in each arm will participate in in-depth qualitative interviews at study completion and will receive $50 for the interview (at 12 months). Additionally, enrolled participants may refer up to four individuals to the study and receive $50 each if randomized. Total possible compensation will be $600.

## Assignment of interventions: allocation

### Sequence generation

Participants (n = 350) will be randomized by permuted block randomization to *C-THR* or non-SSP clinic referral and patient navigation. All randomizations will be stratified by recruitment site (fixed vs. mobile) and baseline OUD diagnosis (OUD vs. no OUD). Randomization will occur with central control using the REDCap program and will be external to the IDEA Miami SSP to assure a robust and unbiased approach.

### Concealment mechanism

Centralized randomization will occur in REDCap and group assignment will be concealed until randomization.

### Implementation

The study coordinator and the research assistant will enroll eligible participants into the trial for random assignment after approval by the study principal investigator. The statistician will encode REDCap with the allocation sequence which will assign treatment condition after the eligibility REDCap form has been completed by the principal investigator.

## Assignment of interventions: blinding

### Who will be blinded

This clinical trial is unblinded. For a robust and unbiased approach, our randomization procedure is strong with central control external to the study site. All data analysis strategies are pre-specified and the finalized data analysis plan will be confirmed prior to data lock. The data analysis will not be blinded since TSB and DJF are study investigators and performing the analysis. TSB and DJF will, however, be blinded to treatment assignment in conducting their analysis until results are finalized.

### Procedure for unblinding if needed

Study is open-label, unblinded.

## Data collection and management

### Plans for assessment and collection of outcomes

Data collection for the efficacy analysis is planned at screening, baseline, and at 3-, 6-, 9- and 12-month follow-up assessments (Table 3). All participants will complete quantitative questionnaires in English and Spanish through the secure REDCap platform at baseline, and at 6- and 12-month follow-up assessments. Measures from NIDA’s Data Harmonization projects, specifically PhenX Toolkit [70], will be included. Assessments will take approximately 1 h and include socio-demographics, SUD treatment history, sexual and injection risk behaviors, substances used by injection and other routes of administration, and healthcare service utilization. Importantly, in pursuit of relevant endpoints important for people with SUD, quality of life measures [71, 72] will be assessed in addition to any changes in syndemic factors such as social determinants of health (e.g., employment, housing, food insecurity, incarceration) and mood (e.g., PHQ-9 [73], BAI [74]). We will use the EuroQol 5D (EQ-5D) to measure health-related quality of life [75, 76]. Stigma and discrimination will be measured using the Substance Use Stigma Mechanisms Scale (SU-SMS) [77], the Group-based Medical Mistrust Scale [78], the PrEP Stigma Scale [79], and Physician–Patient Relationship Scale [80].

**Table 3:**
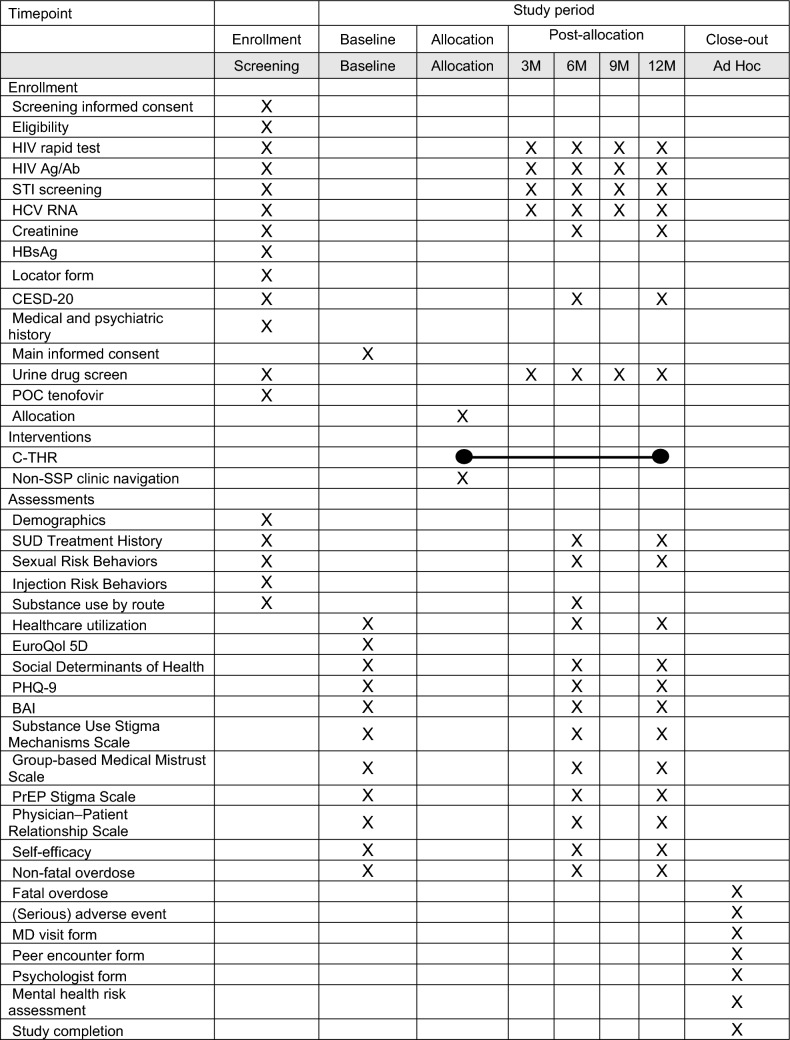
Assessment schedule

All participants will complete laboratory assessments at screening and at 3-, 6-, 9- and 12-month follow-up assessments. At screening, this will include rapid HIV test by fingerstick and point of care urine screens for tenofovir, buprenorphine, and methadone to determine eligibility. Additional tests at screening include fourth generation HIV test, HCV RNA, STI screens, hepatitis serologies, comprehensive metabolic panel, complete blood count and, as indicated, urine pregnancy test. At baseline and at 3-, 6-, 9- and 12-month follow-up assessments, these will include urine drug screen for buprenorphine/norbuprenorphine and methadone/EDDP (methadone metabolite) and fingerstick DBS to test for both the presence and therapeutic levels of TFV-DP related to the primary outcome, as well as HIV Ag/Ab screen, HCV RNA, HIV/HCV rapid tests, and STI screen (including syphilis via blood and gonorrhea/chlamydia via throat swab, rectal swab, and urine). Additionally, participants in the *C-THR* arm will also have a creatinine assessment at 6 and 12 months.

To confirm primary outcome by medical record abstraction, the electronic health record will be quieried for date of injection of (1) cabotegravir, (2) naltrexone or (3) buprenorphine. A cabotegravir injection in the previous 8 weeks will be considered as having met the primary outcome. A naltrexone or buprenorphine injection in the previous 4 weeks will be considered as having met the primary outcome.

The cost and cost-effectiveness analysis will consist of an economic evaluation of *C-THR* versus non-SSP clinic referral and patient navigation from both the healthcare sector perspective and the modified societal perspective. Specifically, the evaluation will focus on budgetary impact and cost-effectiveness to inform application of *C-THR* locally and nationally. This evaluation will follow recommendations of the 2nd Panel on Cost Effectiveness in Health and Medicine [45], Glick et al. [46] and Drummond [47].

Specifically, start-up and ongoing management costs related to the two strategies will be collected using macro-costing (“top down”) and micro-costing (“bottom up”) techniques using a tailored version of the Drug Abuse Treatment Cost Analysis Program (DATCAP) instrument [81], a standardized, customizable tool used to estimate costs of treatment in diverse settings. The macro approach is based on financial information received from the sites, such as invoices and time sheets, and the micro approach is based on semi-structured interviews with site leaders and staff, which will be conducted via site visits, video conferences, and phone calls to capture both accounting and economic costs. Financial information will be supplemented with healthcare utilization data from electronic health records and health system administrative databases and with semi-structured interviews with staff members, which will allow for the identification of other resources used to provide *C-THR* services. To perform a modified societal perspective analysis, data will be collected regarding criminal justice involvement during the trial. The REducing infections related to Drug Use Cost-Effectiveness (REDUCE) model [49] will be adapted and employed to project the long-term clinical and cost-effectiveness of the intervention at preventing incident cases of HIV over the 5-, 10-year, and lifetime horizons.

Finally, the implementation process evaluation will be mixed-methods using the Practical, Robust Implementation and Sustainability Model (PRISM) as the primary conceptual framework for guiding and evaluating the implementation efforts [82]. PRISM emphasizes the importance of multilevel contextual factors that act as drivers of implementation outcomes, such as the organizational and the patient perspective of the intervention, the external environment (i.e., community-level resources) and the implementation and sustainability infrastructure. PRISM was selected in part due to synergy with the RE-AIM evaluation framework [83].

PRISM and RE-AIM will inform both quantitative and qualitative data collection instruments focused on organizational-, provider-, and patient-level domains regarding the intervention based on constructs identified in the PRISM model; the relationship between these contructs and specific RE-AIM outcomes will also be evaluated. This information alongside the RCT will provide data needed to interpret a potential null effect of the intervention as a need for further intervention refinement or a failure of implementation. In addition, in-depth interviews with key informants (n = 20) within the Miami site and system (e.g., SSP staff, trial implementers, study physicians) at two distinct timepoints (at 6-months and trial closeout) will be conducted to assess organizational-level influences on Reach, Implementation, and Maintenance metrics. Additionally, 30 participants will complete qualitative assessments in English or Spanish at 12-month follow-up assessments. Participants will be asked about their experience engaging in the *C-THR* intervention, additional modifications needed to improve the intervention design, and additional strategies that could be used to increase the future reach of the intervention.

Interviews will be used to inform ongoing optimization and adaptations as the trial progresses. In addition to the set interview schedule, a review of trial logbooks and on-site observation data will be used to record strategies used and barriers encountered to enhance our evaluation efforts.

### Plans to promote participant retention and complete follow-up

Participants will be recruited from the IDEA Miami SSP by our racially and ethnically diverse staff that includes peer counselors. Due to the fundamental trust that the SSP has within the community of PWID, we anticipate that utilization of the harm reduction approach of meeting potential participants where they are will support ongoing recruitment into the trial due to the benefits of participation (including telehealth access to a physician, psychologist, counselor). Study staff are experienced and trusted in the community of PWID, and our empowerment of peers as the true experts in PWID health will facilitate recruitment. Providers can remain permanent after trial conclusion since stigmatization continues to be omnipresent in the traditional healthcare system. In our *THR* for PWID with HIV pilot (n = 35), all PWID continued to receive HIV primary care via *THR* after the pilot, even when no longer utilizing syringe services. Since all participants will be recruited via the SSP, the routine exchange of syringes and street outreach will ensure ongoing contact with all participants in the trial. Participants typically visit the program weekly and this ongoing distribution of harm reduction supplies will allow the study team to retain participants in the trial. In CTN-67 CHOICES, of 47 randomized patients with HIV and OUD at the University of Miami site, we retained 45 (96%) at 12-month follow-up. We also plan to use a locator form that we have used in other CTN studies and that will be reviewed by our community advisory board. Based on previous published studies regarding HIV prevention among PWID and to ensure we obtain adequate power to detect an effect, we have powered the trial for 12-month retention rates of 66%.

### Data management

A study-specific data management protocol and standard operating procedures will guide the study team throughout the trial. All data management activities will utilize REDCap, which is secure, HIPAA compliant, and web-based and has real time validation rules at the time of entry. The lead statistician will provide ongoing oversight of data management throughout the study and oversee the data manager in generating regular reports for quality control and data analysis. A study-specific data dictionary will be developed by all members of the research team. Data management reports will be made to the Data and Safety Monitoring Board (DSMB). Cleaned, de-identified data files will be produced for analysis.

### Confidentiality

All data will be kept confidential, electronically locked via password, and available only to authorized staff. The passwords will change periodically and accessible only to specified study staff. Participant data will be identified by an ID number only in the REDCap database. The link between names and ID numbers will be kept separately under password protection that only the site PIs can access*.* Participant case management files (including consents for third party pickup of medications, consents for medication storage, and locator forms) will be locked in a secure file cabinet in a room accessible by keycard and monitored by University of Miami Security.

All participants will be advised that they may decline to answer any study question. These procedures will be implemented to provide study participants with the assurance of confidentiality around sensitive and personal information relating to their mental health, sexual and substance use behaviors, and HIV status. All study personnel working on the project will be trained in human subjects research, good clinical practice, and the importance of strictly respecting participants' rights to confidentiality.

### Plans for collection, laboratory evaluation and storage of biological specimens for genetic or molecular analysis in this trial/future use

The primary and secondary outcomes for objective 1 will be measured with TFV-DP on fingerstick DBS, buprenorphine/norbuprenorphine or methadone/EDDP (methadone metabolite) on UDS, HIV Ag/Ab screen, HCV RNA, HIV/HCV rapid tests, and STI screen (including syphilis via blood and gonorrhea/chlamydia via throat swab, rectal swab and urine). These laboratory assessments will be completed as routine blood, urine, and throat and rectal swab lab tests, sent to a commercial laboratory or Florida Department of Health, and destroyed according to their procedures.

## Statistical methods

### Statistical methods for primary and secondary outcomes

#### Preliminary analyses and missing data

Frequency tables for all variables and measures of central tendency and variability for continuous variables will characterize the sample and be stratified by randomization group (i.e., intervention vs. control). As recommended by CONSORT guidelines (http://www.consort-statement.org/), we will not do statistical tests comparing randomized groups at baseline. Our primary analyses will need to control for any measured characteristics that predict missing data. Thus, in a preliminary analysis we will examine whether any baseline characteristics predict the pattern of missing data observed in the trial. All important predictors from this analysis will be included as control variables in the primary tests of hypotheses. In addition, for our moderator (and mediator) hypotheses, there may be some missing data on self-reported syndemic factors, for example. We will address any incomplete data with multiple imputation [84], full-information maximum likelihood or the expectations-maximization algorithm, all of which make the relatively mild assumption that incomplete data arise from a conditionally random (MAR) mechanism [85–87]. Auxiliary variables will be included to improve efficiency and any potential bias [88, 89]. The proposed analyses will be conducted using validated algorithms in SAS [90] or Mplus [91]. All program code and results will be documented extensively and archived to enable future review, transparency, and results reproducibility.

##### *Hypothesis 1*

*C-THR* will be superior to non-SSP clinic referral and patient navigation in promoting engagement in HIV prevention as measured by the proportion of participants taking PrEP and/or MOUD across follow-up at 3, 6, 9 and 12 months post-randomization.

### Primary inferential analyses to address primary hypothesis of objective 1

We hypothesize that, for those randomized to the *C-THR* arm, the odds of our two co-primary outcomes, engagement in PrEP and engagement in MOUD, will be higher than for those randomized to the non-SSP clinic patient navigation, control arm (Hypothesis 1). Generalized estimating equations (GEE) [92] will be used to perform the proposed primary analysis, which is a planned time-averaged comparison of all post-baseline measurements of the each of these outcomes—PrEP: DBS evidence of tenofovir or cabotegravir injection in the previous 8 weeks by electronic health record abstraction; MOUD: UDS with norbuprenorphine or methadone/EDDP; or naltrexone or buprenorphine injection in previous 4 weeks by electronic health record abstraction—across the *C-THR* and control groups to test primary Hypothesis 1. Alpha will be set at 0.025 for this planned comparison to control the Type-1 error across these two co-primary outcomes. In each model, recruitment site (fixed site vs. mobile site) and OUD diagnosis (yes vs. no) will be included in these analyses as required by the stratified randomized design to obtain unbiased results. If the overall test of the intervention is statistically significant, individual contrasts for differences between the conditions at each of the times of follow-up will be performed.

Though GEE estimates are consistent even if the correlation structure is misspecified, GEE's statistical efficiency improves as the working correlation structure more closely approximates the actual correlation structure [92], so various correlation structures suitable for the study's design will be considered (e.g., exchangeable, autocorrelated, *m*-dependent, unstructured) [93]. The QIC statistic will be used to select the final correlation structure [94]. Recruitment site (fixed site vs. mobile site) and OUD diagnosis (yes vs. no) will be included in all models as required by the stratified randomized design to obtain unbiased results [95]. Robust standard errors will be used to obtain correct inferences even if the chosen correlation structure remains slightly mis-specified. All analyses will include outlier and influential case screening via computation of GEE-based residual analysis, including leverage, DFBeta, and Cook’s D statistics [96]. If outliers are found, results will be reported with and without outliers included.

### Data analysis plan and statistical procedures to address secondary hypotheses of objective 1

We have proposed several secondary hypotheses related to the *C-THR* intervention. The secondary analyses will use exactly the same preliminary analysis and missing data strategy as Hypothesis 1. In secondary analyses, we will examine the distributions of syringe coverage, PrEP adherence, HCV treatment engagement, HCV cure, STI treatment uptake, and number of injection related harms and assess time to first injection-related harm between the intervention and control groups. GEE [92] will be used to perform the proposed secondary analysis, which is a planned time-averaged comparison of all post-baseline measurements of the each of these outcomes. Statistical procedures for these secondary hypotheses will follow the same strategy used for the primary hypothesis. The alpha will be set at 0.05 for each these secondary hypotheses. In each model, recruitment site (fixed site vs. mobile site) and OUD diagnosis (yes vs. no) will be included in these analyses as required by the stratified randomized design to obtain unbiased results. To maximize rigor, appropriate diagnostics including outlier and influential case screening will be examined for each model. If outliers are found, results will be reported with and without outliers included.

The count of harms will be assessed in a similar fashion as the analysis of the secondary hypotheses except that the distribution will be either a Poisson or Negative Binomial (the Negative Binomial will be used if overdispersion is present) and the link function will be log. For time to first harm, the comparison will be made using the logrank test and Kaplan–Meier estimates of the survival curve.

### Sex as a biological variable

We will examine the primary hypothesis and secondary hypotheses with respect to difference by sex. In particular, the primary analysis method will be used and a sex by treatment interaction in the time-averaged odds of engagement in HIV prevention will be added to the model to test whether there are differential treatment effects by sex. With the expected 120 or 1/3 of the sample women, there is reasonable power to uncover a difference.

### Interim analyses

If study recruitment fails to meet quotas and the original target sample size appears unlikely to be achieved, investigators will propose an updated target sample size and conduct a futility analysis. Conditional power will be calculated based on the treatment effect size observed in the current data and “information fraction” consistent with the updated target sample size. This analysis indicates the likelihood of finding a significant effect if trends in the current data continue and the updated sample size target is met. If conditional power under the updated sample size fails to meet a pre-specified threshold of 0.5, the stopping rule will be considered satisfied. If, on the other hand, conditional power is high, the trial is likely to meet its primary outcome even with the reduced sample size. The DSMB will use this information to guide its recommendation to continue or discontinue the trial.

### Methods for additional analyses (e.g. subgroup analyses)

We will use the following data analysis plan and statistical procedures to address mediation and moderation.

### Moderation analyses

*C-THR* will buffer the impact of syndemic factors (e.g., unstable housing, food insecurity) on hypothesized outcomes. The GEE model will be used to examine the relationship between syndemic factors and the hypothesized outcomes (see above). Then an interaction between the syndemic factors and treatment assignment will test whether the impact of syndemic factors were lessened in *C-THR*.

### Mediation analyses

The impact of *C-THR* on our hypothesized outcomes will be mediated through reductions in stigma. To maximize rigor, these analyses will not be performed with classical multiple regression-based mediation techniques such as the Baron and Kenny causal steps approach [97]. Instead, mediation analyses will be conducted using structural equation modeling (SEM) and bootstrapped tests of significance of the product of coefficients (a*b, where a is the path coefficient from the intervention to mediator, and b is the path coefficient from mediator to outcome). We will use M*plus* [91] to perform mediation analyses because it unites SEM with causal inference-based mediation methods in the same analysis platform [98]. Alpha will be set at 0.05 for all hypothesis tests in these exploratory analyses.

### Methods in analysis to handle protocol non-adherence and any statistical methods to handle missing data

We will address any incomplete data with on of three approaches: multiple imputation [99], full-information maximum likelihood [87] or methods which include the expectations-maximization algorithm [86]. All of these approaches make the relatively mild assumption that incomplete data arise from a conditionally random (MAR) mechanism [100].

### Plans to give access to the full protocol, participant level-data and statistical code

We will provide de-identified data to interested investigators one year after publishing the primary outcome paper. After obtaining IRB approvals for planned analyses, and any needed data sharing plans, de-identified data will be sent.

## Oversight and management

### Composition of the coordinating center and trial steering committee

The data monitoring and management procedures will be established to maintain active, clear communication at the study site. Specifically, the lead study investigators will hold weekly zoom meetings with study staff to direct study activities. This meeting schedule will be maintained throughout all years of the project. These meetings will provide general training and address consistency of procedures as well as problems and challenges. Topics will include issues of recruitment and retention, data collection/management/analysis, budget, and protocol fidelity. The study physicians and psychologist will supervise and train harm reduction counselors in the C-*THR* intervention. The statistician will generate regular quality control reports for the sites and make real-time corrections.

The *C*-*THR* manual was adapted from the *THR* manual, which was developed by Drs. Tookes Bartholomew, Serota, and Suarez in conjunction with harm reduction counselors. Regular zoom meetings to support harm reduction counselors in the delivery of the intervention will occur. Study investigators will assess the extent to which the intervention was implemented in a manner that is maximally consistent with the intent of the intervention manual though periodic fidelity monitoring of intervention session audio recordings.

Data forms will undergo a rigorous systematic editing process prior to entry into the REDCap database. The study statistician will routinely evaluate the data and discuss any problems with study staff and investigators at the weekly team meetings. Data management formal reports on record status across the three following domains will be employed: entered, verified, and edited. These reports will be evaluated once monthly during the team meeting. To help ensure data protection, back-up copies will be automatically generated by our computer systems. Data collected from study assessments and questionnaires will be entered directly into REDCap. Confidentiality will be assured as participants will be identified on all study materials only by participant number, visit number, and date of visit. By recording the study data in this manner, the information can be considered “de-identified,” and therefore, compliant with the Standards for Privacy of Individually Identifiable Health Information (“Privacy Rule”) of the Health Insurance Portability Act of 1996 (“HIPAA”).

### Composition of the data monitoring committee, its role and reporting structure

To fulfill its mission of ensuring the safety and integrity of the study, it is necessary that the DSMB be comprised of members who possess a high degree of competence and experience, as well as the ability to function independently of all other parties involved in the study. The DSMB members will function free of the career and financial interests of its members and will consist of three members with experience in conducting clinical intervention research on HIV prevention, expertise in biostatistics, and a thorough knowledge of clinical trial ethics and human subject protection issues.

The DSMB will meet annually by zoom conference and will be updated semi-annually by report. At the yearly meeting members will review randomization data as well as adverse events. The following serious adverse events will be reported within 24 h if determined to be related to the study: deaths, hospitalizations, fatal overdoses, and psychiatric hospitalizations.

### Adverse event reporting and harms

It is unlikely that participants will be at substantial risk for harm as a result of study participation. The study involves facilitating access to HIV prevention medications, and the risks of PrEP and MOUD are the same as if they were prescribed as part of standard of care. They are described herein because they will be recorded as study related adverse events and include outcome measures. Participants that are prescribed PrEP and/or buprenorphine will complete medical monitoring for potential side effects as part of their clinical care. Any drug related toxicities (e.g., elevated creatinine) or injection site reaction (e.g., cabotegravir, naltrexone) will be monitored by the study team. Buprenorphine has a risk of overdose when combined with benzodiazepines or alcohol. Participants will be advised of this risk as is standard medical care. The risk of overdose from buprenorphine is small compared to the risk of intravenous fentanyl injection so the benefits outweigh risks. Additionally, participants may find some of the questions asked in the questionnaire to be upsetting. Study labs will be conducted routinely and may cause slight discomfort. There is always the potential risk of loss of confidentiality, but procedures are in place to prevent this potential risk. Using telehealth, it is possible that a sign or symptom could be missed. Peer counselors will assist the physician and psychologist in physical diagnosis. The physician will conduct a thorough review of systems. If necessary, in-person exams will be available at IDEA.

Participants will be queried for new potential adverse events at each research visit after screening informed consent. In the *C-THR* arm, peer counselors will receive training to address medication non-adherence, sexual and injection risk taking, and substance use behaviors. The clinical psychologist will be available on-demand for any participant in either study arm experiencing severe emotional distress, suicidality, or homicidality. He will be backed up by the study clinicians who have a rotating on-demand call schedule. Additionally, the SSP has licensed mental health counselors on staff in the case of mental health crisis. During the behavioral assessments, the REDCap survey will automatically provide an alert for a score on the Center for Epidemiologic Studies Depression Scale (CES-D) that suggests that the person is experiencing severe depressive symptoms. Any necessary linkage to emergency services will be provided. With respect to blood draws, each of these procedures will be carried out by phlebotomists trained to work with PWID or study physicians to minimize the accidental injury or discomfort to the participants. Participants who experience harm as a result of these procedures will receive first aid from study staff and referral to medical professional if needed.

### Frequency and plans for auditing trial conduct

As a clinical trial in a harm reduction setting, the CHARIOT trial has a rigorous quality assurance plan. Periodic ‘interim’ site monitoring visits will be conducted approximately every 12 weeks. The purpose of these is to review study documentation for accuracy and completeness, to conduct source-to-database comparisons for data quality, and to monitor compliance with the study protocol and procedures. Interim visits may be conducted on-site or remotely. Site performance rates (i.e., recruitment and retention) will be reviewed on a monthly basis and feedback will be provided to the study site regarding their success in meeting performance targets. The scope of the visits will include reviewing the following documentation for 100% of consented participants: (1) informed consent forms, HIPAA forms, and medical records releases; (2) eligibility forms/documentation; (3) electronic data capture-to-source verification for data points related to co-primary outcomes at 3, 6, 9 and 12 months post baseline; (4) adverse event/serious adverse event documentation; and (5) protocol deviation documentation, as applicable.

The external Quality Assurance (QA) monitor will provide consultation to the study Lead Team on quality-related matters including recommendations for quality controls that can be built into the procedures of the study (e.g., visit checklists/progress notes and study implementation logs; guidance regarding Regulatory File set-up; support on the evaluation of protocol deviations and determination of corrective actions; recommendations for providing regular site-level feedback on performance targets). The QA monitor will participate in the training of study teams, delivering training on good documentation practices, conduct internal quality control as part of daily study activities and provide refresher training on QA-related matters to site teams (PIs, study coordinators, peers) as needed. The QA monitor will provide on-site and/or remote monitoring at: (1) site initiation to assess site readiness for launch; (2) periodically throughout study implementation to assess data quality and protocol compliance; and (3) at study closeout and will submit reports of monitoring findings and track all findings to resolution. Finally, the QA monitor will attend study calls to remain informed on study progress and milestones, as well as specific implementation challenges faced by the site and provide QA updates and announcements.

### Plans for communicating important protocol amendments to relevant parties (e.g. trial participants, ethical committees)

Any amendments to the protocol or consent will be approved by the IRB prior to implementation. Participants will be reconsented if the consent is amended to include new risk information (e.g., changes in the anticipated risks/benefits of participation) or other information that involves significant changes to the procedures of the study.

### Dissemination plans

Community engagement is a central component of our work in harm reduction. Accordingly, we have included PWID who are actively using substances or who are in recovery during our planning of the intervention and will continue to include them throughout the conduct of the trial. The community will inform us of the acceptability of our recruitment and retention efforts and any changes that might be needed to the protocols during the start-up period. PWID will lead our active community dissemination as findings from this trial emerge. Together with our peer staff, as founders and leaders of the “home base” for PWID, the study team will stay closely connected to the community throughout the duration of the study. We will participate in community advisory board (CAB) meetings through the University of Miami Center for AIDS Research (CFAR) CAB, the University of Miami Center for HIV and Research in Mental Health (CHARM) AIDS Research Center (ARC) CAB, and the Florida Harm Reduction Collective CAB. We will solicit input from the CABs and PWID as to how to best stay engaged with the community in terms of dissemination of findings.

We registered the trial on ClinicalTrials.gov to ensure that results are submitted according to the required policies in a timely manner and that registration and results information remain up to date. We registered the study before the first subject randomization and will update every 12 months at minimum. Summary results will be submitted less than one year after the trial’s completion. Through the University of Miami login, the principal investigators will manage the ClinicalTrials.gov study updates. We will include information about the posting of the results on ClinicalTrials.gov on the study consent.

In addition to the main outcome paper, we plan to publish multiple manuscripts from the study in peer-reviewed journals. We expect to have a separate manuscript regarding the economic evaluation and cost-effectiveness and the long-term projected clinical impacts and costs from our simulation modeling. We expect to have additional manuscripts regarding implementation procees and outcomes evaluation from the trial. We also expect to publish at least one baseline manuscript characterizing the sample and focusing on the prevalence and impact of syndemics in the population, and one manuscript detailing the intervention.

After the trial is completed, we plan to present the results of the study at scientific conferences and to state and national healthcare administrators and policymakers. Florida Department of Health and Florida Department of Children and Families consult us routinely in the statewide expansion of harm reduction services. The National Harm Reduction Coalition, AIDS United, the National Association of State and Territorial AIDS Directors, the Drug Policy Alliance, and the North American Syringe Exchange Network will partner with us in development of technical assistance and best practices to be disseminated to harm reduction programs immediately and routinely throughout the trial due to our “no time to wait” approach in the unending HIV and overdose crises in the community. We plan to present findings at their conferences and provide consultation to these national organizations in their development of technical assistance packages, as well as provide insight into lessons learned in implementation of *C-THR*. Partnering with these organizations, we plan to leverage objective 3 in the development of an implementation toolkit for immediate dissemination. Finally, we will continue to provide consultation on lessons learned and best practices to the Centers for Disease Control and Prevention, the White House Office of National AIDS Policy, the White House Office of National Drug Control Policy, the Substance Abuse and Mental Health Services Administration, and the Department of Health and Human Services as key stakeholders in their Ending the HIV Epidemic and National Drug Control Strategies.

## Discussion

The CHARIOT trial comes at a critical time when IDU continues to contribute significantly to the ongoing HIV epidemic in the US, including outbreaks in multiple jurisdictions [3–8]. At the same time, SSPs are expanding into new locations and many are providing new medical services, particularly MOUD under the new Drug Enforcement Administration rules [101–103, 32, 104–108]. Built upon the decades of evidence of the effectiveness of SSPs in prevention of HIV, *C-THR* offers a unique opportunity to bring comprehensive HIV prevention to PWID on their own terms in a true harm reduction approach—meeting them where they are, both mentally and physically, in a respectful environment, overcoming the intransigent stigma [109, 110] that permeates the tradititional healthcare system [111].

In our previous work interviewing PWID to determine how to best deliver comprehensive HIV prevention to this community, our SSP participants advocated for a “one-stop shop” where PrEP and MOUD would be delivered via an SSP with evidence-based harm reduction interventions of syringes and naloxone [112]. In our pilot (n = 109), we adapted the *THR* intervention to offer low barrier, free buprenorphine at the SSP with promising results of 59% retention at 3 months [113]. Building on this work, we now seek to adapt and test the *C-THR* intervention for PWID to address their HIV prevention and substance use needs more fully.

There have been challenges in launching this transformative CHARIOT Trial, mostly due to the study setting. Florida’s sobriety requirements previously limited access to DAA in PWID. At the strong recommendation of our study team to Florida’s Agency for Healthcare Administration, sobriety guidelines have been removed for DAA [114]. Florida Medicaid previously had strict abstinence guidelines for buprenorphine treatment but now, again at the urging of our study team, Medicaid utilizes an auto-prior authorization for buprenorphine [115]. To facilitate access to PrEP and DAA, we have enrolled in the Health Resources and Services Administration 340b program for discounted medications. Access to buprenorphine will also be supported by the Florida Department of Children and Families (SAMHSA recepient) low barrier buprenorphine program at IDEA Miami SSP funded through State Opioid Response funds. Additionally, while Florida is not a Medicaid expansion state, peer counselors will assist participants in open enrollment under the Affordable Care Act. We also recognize the unique implementation context (an academic-medical center based SSP) of this trial and its impact of future scalability. Many SSPs have limited resources and no connection to the traditional healthcare system which could make scalability of the *C-THR* model to other SSPs more difficult.

The CHARIOT trial is a pragmatic clinical trial [116] that has the potential to have a major impact on PWID health. We plan to transform the way we deliver comprehensive HIV prevention to PWID by testing an innovative, telehealth-enhanced care model implemented in an SSP setting. Consistent with harm reduction principles, our approach to *C-THR* has been informed by PWID both through qualitative interviews and through intervention design with staff peers. The cost-effectiveness analyses, simulation modeling, and mixed-methods implementation evaluation will provide compelling data on the broader adoption and sustainability of the *C-THR* care model. In pursuit of a one-stop shop for comprehensive HIV prevention, *C-THR* could make Ending the HIV Epidemic an attainable reality for PWID.

## Data Availability

Any data required to support the protocol can be supplied on request to the PIs.

## Abbreviations

ARC: AIDS Research Center
CAB: Community advisory board
CES-D: Center for Epidemiologic Studies Depression Scale
CFAR: Center for AIDS Research
CHARIOT: Comprehensive HIV and Harm Prevention Via Telehealth
CHARM: Center for HIV and Research in Mental Health
C-THR: Comprehensive Tele-Harm Reduction
DAA: Direct-acting antiviral
DATCAP: Drug Abuse Treatment Cost Analysis Program
DBS: Dried blood spot
DSMB: Data and Safety Monitoring Board
EDDP: 2-Ethylene-1,5-dimethyl-3,3-diphenylpyrrolidine
FQHC: Federally qualified health center
GEE: Generalized estimating equations
HCV: Hepatitis C virus
HIV: Human immunodeficiency virus
ICER: Incremental cost-effectiveness ratio
IDEA: Infectious Disease Elimination Act
IDU: Injection drug use
IRB: Institutional review board
MI: Motivational interviewing
MOUD: Medications for opioid use disorder
OUD: Opioid use disorder
PrEP: Pre-exposure prophylaxis
PWID: People who inject drugs
QA: Quality assurance
QALY: Quality-adjusted life year
RCT: Randomized controlled trial
REDUCE: REducing infections related to Drug Use Cost-Effectiveness
SAMHSA: Substance Abuse and Mental Health Services Administration
SEM: Structural equation modeling
SPIRIT: Standard Protocol Items: Recommendations for Interventional Trials
SSP: Syringe services program
SSTI: Skin and soft tissue infection
SUD: Substance use disorder
TFV-DP: Tenofovir diphosphate
THR: Tele-Harm Reduction
UDS: Urine drug screen
US: United States
